# CollaborationViz: Interactive Visual Exploration of Biomedical Research Collaboration Networks

**DOI:** 10.1371/journal.pone.0111928

**Published:** 2014-11-18

**Authors:** Jiang Bian, Mengjun Xie, Teresa J. Hudson, Hari Eswaran, Mathias Brochhausen, Josh Hanna, William R. Hogan

**Affiliations:** 1 Division of Biomedical Informatics, University of Arkansas for Medical Sciences, Little Rock, AR 72205, United States of America; 2 Department of Computer Science, University of Arkansas at Little Rock, Little Rock, AR 72204, United States of America; 3 Department of Psychiatry and Behavioral Sciences, University of Arkansas for Medical Sciences, Little Rock, AR 72205, United States of America; 4 Department of Obstetrics & Gynecology Research, University of Arkansas for Medical Sciences, Little Rock, AR 72205, United States of America; 5 Department of Veterans Affairs HSR&D Center for Mental Healthcare and Outcomes Research, Central Arkansas Veterans Healthcare System, Little Rock, AR 722205, United States of America; 6 Clinical and Translational Science Informatics and Technology, University of Florida, Gainesville, FL 32610, United States of America; 7 Department of Health Outcomes & Policy, University of Florida, Gainesville, FL 32610, United States of America; 8 Clinical and Translational Science Institute, University of Florida, Gainesville, FL 32610, United States of America; Tianjin University, China

## Abstract

Social network analysis (SNA) helps us understand patterns of interaction between social entities. A number of SNA studies have shed light on the characteristics of research collaboration networks (RCNs). Especially, in the Clinical Translational Science Award (CTSA) community, SNA provides us a set of effective tools to quantitatively assess research collaborations and the impact of CTSA. However, descriptive network statistics are difficult for non-experts to understand. In this article, we present our experiences of building meaningful network visualizations to facilitate a series of visual analysis tasks. The basis of our design is multidimensional, visual aggregation of network dynamics. The resulting visualizations can help uncover hidden structures in the networks, elicit new observations of the network dynamics, compare different investigators and investigator groups, determine critical factors to the network evolution, and help direct further analyses. We applied our visualization techniques to explore the biomedical RCNs at the University of Arkansas for Medical Sciences – a CTSA institution. And, we created CollaborationViz, an open-source visual analytical tool to help network researchers and administration apprehend the network dynamics of research collaborations through interactive visualization.

## Introduction

Clinical translational science embraces inter-disciplinary collaborations. One of the key objectives of the Clinical Translational Science Award (CTSA) is to promote cross-disciplinary collaborations that can accelerate the translation and application of biomedical research discoveries into clinical settings. To better understand, facilitate, and direct clinical and translational research efforts, it is essential to analytically assess the quality and efficiency of existing research collaborations in a CTSA institution and promptly identify those potential collaborations that are more likely to be productive and make significant impact. Social network analysis (SNA) has been deemed as an effective tool to assess inter- and intra-institution research collaborations in the CTSA community [1]. Studying different collaborative relationships (e.g., co-authorships in scientific publication and collaborations on grants), a number of studies on research collaboration networks (RCNs) [2]–[8] have provided insights into the networks' topological characteristics and the network dynamics of research collaborations. For example, using various network centrality measures [8], we can identify key entities/components of the collaboration network, which enables us to allocate resources strategically and therefore boost the overall network efficiency, e.g., attract new investigators to join the network and spawn new collaborations.

Although quantitative metrics of RCNs are valuable, the interpretations of descriptive network statistics are difficult for non-experts. Visualization of a RCN, e.g., through a graph where nodes in the graph represent social entities and links among them indicate their interactions, is beneficial to a layperson to understand its topology and dynamics. Visualization has been shown effective to present large amount of information and to stimulate visual thinking. And, visualizing social networks (and network visualization in general) has a rich history [9]–[12]. However, the majority of literature on social network visualization is based on static graph drawing. And most of the visualization tools used by social network analysts focus predominantly on automatic graph layout algorithms. Many SNA studies leverage one of the general-purpose network analysis toolkits such as iGraph [13], NetworkX [14] and Pajek [15] that provides some basic visualization capabilities. However, due to the limitation of those tools, often only static visualization of the networks are presented in those SNA studies. Our goal in this study is to create an interactive visualization platform that can support a variety of social network analysis tasks pertaining to studying collaborative research relationships. Interactive network visualization techniques can reinforce human recognition and have a profound impact on how best we can represent, analyze, and communicate network data.

In this paper, we present our experiences in exploring various network visualization techniques to create CollaborationViz, an open-source web-based informative and interactive visual analytical tool for studying biomedical RCNs. Specifically, we demonstrate CollaborationViz through analyzing network dynamics and characteristics of the biomedical RCN at the University of Arkansas for Medical Sciences (UAMS) – a CTSA institution. All the resources including the source code of CollaborationViz, the scripts of our network analyses and the anonymized network data can be found at https://github.com/bianjiang/rcna. While in this paper we use a particular dataset to present our work, CollabrationViz supports a set of visual analysis tasks applicable to networks in general and may be adopted by other exploratory visual analysis systems.

## Methods and Technologies

### Dataset and social network analysis of biomedical research collaboration

The biomedical research collaboration networks we study are unique in that those RCNs are formed based on collaborative research grants rather than publication co-authorships [8]. The Office for Research and Sponsored Programs (ORSP) at UAMS uses an in-house developed software system to track detailed information of research grants such as the requested budget amount, the budget start and end dates, the funding agencies, as well as investigators and their roles on each grant. Table 1 shows the statistics of the research grant data we have obtained from the ORSP. Our dataset included all grants that were awarded from 2006 to 2012 (fiscal years). We use these meta-data of grants to construct seven RCNs for each fiscal year from 2006 to 2012, and two aggregated RCNs (one spanning four fiscal years from 2006 to 2009 and the other spanning three years from 2010 to 2012). Besides the ORSP, we also used data collected by the Translational Research Institute (TRI, UAMS) to identify investigators that are supported by the CTSA program at UAMS. The TRI supports all CTSA activities at UAMS since July 2009.

**Table 1:** Statistics of the research grants dataset at the University of Arkansas for Medical Sciences.

Fiscal Year	Number ofAwarded Grants	Number ofInvestigators	Number of CTSAInvestigators^1^	Number of CTSASupported Investigators
2006	477	326	N/A	N/A
2007	479	409	N/A	N/A
2008	601	469	N/A	N/A
2009	516	414	N/A	N/A
**2006–2009**	**2073**	**759**	**N/A**	**N/A**
2010	603	431	34	114
2011	538	443	26	115
2012	549	434	23	322
**2010–2012**	**1690**	**650**	**34**	**551**

1 The CTSA at UAMS started on July 14th, 2009. The “Number of CTSA Investigators” and “Number of CTSA Supported Investigators” are not applicable to fiscal years from 2006 to 2009.

We formalize a biomedical RCN as an ***undirected weighted graph*** to reflect the degree of collaboration, i.e., 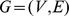, where each investigator is represented by a vertex or node 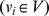, and the collaborative relationship between two investigators (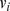 and 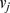) is evident by an edge or link between the two nodes 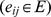. The weight (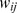) of an edge (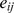) is the number of research grants the two investigators have collaborated on during the time period of interest. Many previous studies on scientific collaborations [2]–[6] model a RCN as a binary network, where an edge is either present or not. In real world, however, the strength of the collaborative ties among different pairs of investigators may vary. One tends to feel more comfortable to work with existing collaborators rather than finding new peers. Therefore, our graph model incorporates non-binary edge weights in network generation and adopts the number of collaborated grants to indicate the extent of collaboration. Clearly, this is a rough approximation as some investigators spend more time than other investigators on the same grant. However, in the absence of other data, using the number of collaborated grants to indicate the strength of the social tie between two investigators is a reasonable approximation [22].

We studied a variety of network characteristic measures pertaining to RCNs, including clustering coefficient, characteristic path length, and number of disjointed components, once a biomedical RCN is generated. In our previous social network analysis of the biomedical RCNs at UAMS [8], we have investigated the effectiveness of the CTSA program and its impact on promoting collaborative research within an institution by observing the temporal evolution of those measures prior to and after the CTSA program at UAMS. Further, we can identify “influential” (or “important”) investigators in the RCNs (in terms of network topology) based on four different network centrality measures–degree, betweenness, closeness, and eigenvector centralities–of the nodes. We have obtained quantitative evidence that the biomedical RCN at UAMS is moving towards favoring cross-disciplinary research after the CTSA award with the help of the diversity measure. Last but not least, we have created a collaboration recommendation model leveraging the random walk with restart (RWR) algorithm for suggesting potential new collaborations. The benchmarks of our recommendation method on the RCNs of UAMS show promising results.

### Modern Web technologies for interactive network visualization

Although static graphs are useful in presenting network structures, they limit the amount of information that can be conveyed and always present the network from a fixed perspective. To deepen our understanding of RCNs and assist nontechnical users in comprehending important network metrics and their implications, we created CollaborationViz, a web-based interactive network visual analytics tool. CollaborationViz is built using a number of cutting-edge web-based visualization technologies, especially the Scalable Vector Graphics (SVG)–a language for building rich graphical content [16], d3.js–a JavaScript library for manipulating SVG objects [17], and Bootstrap–a front-end Web development framework [18]. Network data are stored in JavaScript Object Notation (JSON), a lightweight web-friendly data-store and data-interchange format [19].

## Results

**Figure 1:**
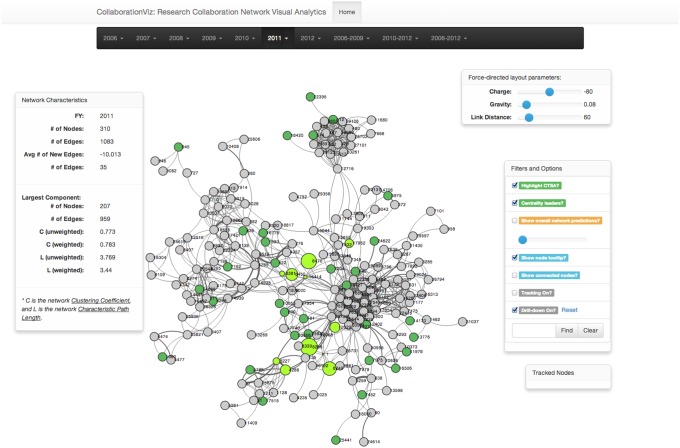
The main interface of CollaborationViz, an interactive visual analytical tool for exploration of biomedical research collaboration networks.

An important goal of our study on RCNs is to provide a set of analytical tools for nontechnical biomedical researchers and administration to understand the nature and evolution of collaboration. As interactive visualization is direct, informative, and user friendly for a person to apprehend data and derive accurate observations and useful insights, CollaborationViz has been created to not only better disseminate the results of our network analyses on biomedical RCNs, but also to support visual analytics. Figure 1 illustrates the main interface of CollaborationViz. Based on our previous study on UAMS’s RCN [8], our design of CollaborationViz starts by considering an analysis process to support exploration and assessment of a research collaboration network with respect to the following objectives: (1) representing collaboration networks in a meaningful format (e.g., a force-directed graph layout); (2) visualizing the strength of the collaborative relationships; (3) visualizing and tracking global and individual changes over time; (4) emphasizing relative importance and possible correlation between nodes (investigators); and (5) demonstrating the predictive power of our collaboration recommendation model. A live demo of CollaborationViz can be found at http://bianjiang.github.io/rcna/.

### Informative representations and interactive network visualization

CollaborationViz provides an informative and intuitive user interface with theoretically-motivated abstractions for nontechnical users to interact with and study a collaboration network. As shown in Figure 1, each circle (node) represents an investigator in the collaboration network, and a curved line connecting two nodes indicates the collaborative relationship between the two investigators. Nodes are colored to distinguish CTSA (green) supported investigators from non-CTSA (gray) supported investigators. The strength of collaboration between two investigators is represented by the thickness of line.

To realize vivid, accurate, interactive representation, networks in CollaborationViz are rendered using a physically-based force-directed graph layout [17]. We can consider the network as a particle system, and the force-directed graph layout in d3.js implements the position Verlet integration to determine moving trajectories of nodes (particles) in the network. Typically, in implementations of particle systems, each particle has two main variables–its position 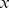 and its velocity 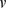. Vertlet integration is a velocity-less schema, where we only store the current position 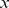 and its previous position 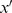 of each particle. The velocity can be implicitly computed and consequently it is easier to keep velocity and position in sync, which makes the simulation more stable [23]. Further, the physically-based model not only considers repulsive charge forces that spread nodes evenly on the canvas, but also takes into account the gravity forces that keep nodes centered in the visible area and avoid expulsion of isolated components [25]. One immediate benefit of using a force-directed graph layout for rendering networks is the clustering effect that manifests. A cluster of nodes that are highly connected will naturally be grouped together because of the gravity forces. For more details, Kobourov has an excellent review article on force-directed graph drawing algorithms that provides more technical background of the implementations [24].

One of the goals in CollaborationViz is to integrate various contextual information along with the node-edge graph. Network characteristics such as the numbers of nodes, edges and isolated components of a network are readily available along with the network diagram. A user can hover her mouse cursor over each node to see the node's local network characteristics including its local clustering coefficient and four centrality measures (degree, betweenness, closeness and eigenvector centralities). These network topological features help understand the structure of the overall network as well as the importance and position of each investigator in the RCN. A user can also drag a node to a different position and the nodes that are incident to this node will also be repositioned according to the physically-based graph rendering model. The parameters (i.e., charge, gravity, and link distance) of the force-directed layout algorithm are adjusted and the changes will be reflected immediately on the canvas.

### Temporal evolution of research collaboration networks

The ability to visually exam the research collaboration networks at an institution is crucial to the understanding of the evolution of the network dynamics, therefore the development direction of the research environment under study. CollaborationViz not only provides a timeline that shows snapshots of the overall network of interest at different time periods, but also gives the ability to track individual investigator’s development in the network across different time spans.

**Figure 2:**
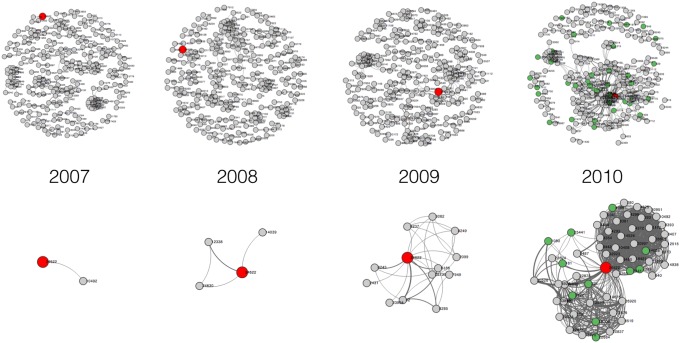
Temporal evolution of an investigator in the research collaboration network at UAMS.

Figure 2 shows a use case of using CollaborationViz to explore the advancement of an investigator’s collaborative relationships within UAMS’s RCNs from 2007 to 2010. The chosen investigator is one of many who have received the TRI pilot awards, which was developed as part of the CTSA program at UAMS to “stimulate and solidify new, innovative research collaborations and promote high-quality translational research”. In Figure 2, the investigator of interest is highlighted in red. The top part of Figure 2 shows four snapshots of the RCN at UAMS from 2007 to 2010– one of each year, and gives us a sense of the relative positions of the particular investigator in the network; while the bottom figures present a focused view of the investigator’s immediate-connections and their changes over the four year, respectively. Through analyzing these figures, we can make the following observations. In 2007, the investigator only collaborated with researchers in an isolated small group (four investigators). In 2008, the size of the group and the number of internal connections increased; however, the group was still disconnected from other parts of the network and the particular investigator still had very few collaborations. In 2009, in preparation of the CTSA program, this group eventually made connections to the largest component (i.e., connected subgraph) of the network. We can easily see in the 2009 graph in Figure 2 that this investigator became a bridge connecting different small clusters. In 2010, the first year after the CTSA, the investigator was drawn towards the center of the network, and her “influence” in the network increased drastically. Moreover, these visual patterns echo our quantitative social network analysis of the investigator’s collaboration network. Positive changes of the investigator’s network characteristics also suggest her increased productivity in research collaborations from 2007 to 2010. For example, the degree of her node in the network increased from 1 in 2007 to 98 in 2010. Furthermore, all of the four centrality measures of this investigator had increased. In particular, the closeness centrality had risen from 1.007 in 2007 to 3.664 in 2010, which coincides with our visual analysis that her position in the RCN became more “central” from 2007 to 2010. Many of other TRI pilot awardees exhibit similar temporal evolutions in network dynamics with increased degree of collaboration and became more “influential” in the network after the awards. Further, we also examined non-CTSA supported investigators’ network developments within the UAMS RCN, and found that their collaboration circles (collaborative relationships) were less developed during the same time period comparing to the CTSA-supported group. These findings are consistent with our previous quantitative analyses [8] which suggest that the CTSA program has a positive effect in promoting research collaboration and such effect is more evident within the group of investigators who are supported by the CTSA.

### Modeling influence in a social network through centrality measures

**Figure 3:**
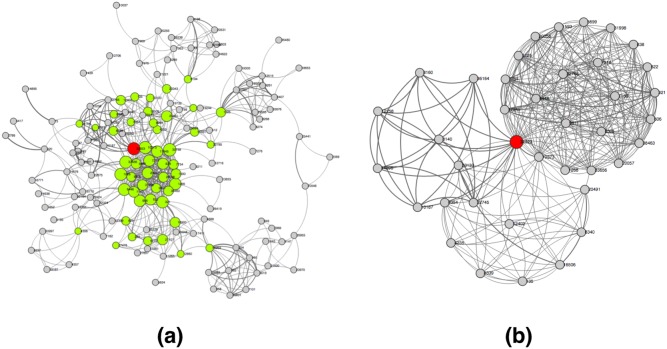
Visualizing “centrality leaders”: Figure 3 (a) demonstrates a visualization of the identified “centrality leaders” and their relative “importance” to the network based on UAMS’s 2012 research collaboration network; and Figure 3 (b) zooms in to one of the centrality leaders and shows her immediate collaborative relationships.

In network analysis, a variety of centrality measures are used to determine the relative importance of a node in the network. However, each centrality measure defines the meaning of importance from a different perspective [26]. Within the context of research collaboration network, centrality measures of an investigator can be interpreted as how influential or important the person is with respect to the structure of the network. To identify influential nodes in a comprehensive manner, we investigated four widely used network centrality measures–degree centrality, betweenness, closeness, and eigenvector centrality [27]–to rank investigators’ relative influence (or importance, contribution) and combine multiple rankings of nodes using rank aggregation techniques [28]. An influential investigator with a high consensus ranking is called a centrality leader who affects others in ways such as propagating an idea or an advertisement across the network. CollaborationViz visualizes a node’s relative influence through adjusting the size of each node according to its ranking of relative influence in the RCN. Combined with other visual analytical tools in CollaborationViz, we can easily identify, analyze and reason with investigators’ relative importance in the collaboration network. Figure 3 (a) demonstrates a visualization of ranking investigators’ relative importance in CollaborationViz based on UAMS’s 2012 RCN. Further, as shown in Figure 3 (b), CollaborationViz gives us the ability to drill down to a specific centrality leader (id: 32923) and it is obvious that this investigator is not only highly connected (a high degree node) but also acts as a hub connecting three communities in the network. Such observations are hard to make and comprehend through a quantitative network analysis, but self-evident in CollaborationViz through novel visualizations.

### Exploration and interaction through highlighting, filtering and visual overlays

**Figure 4:**
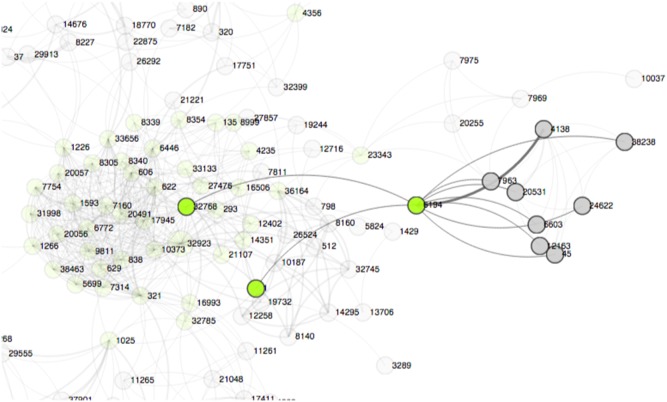
Using transparency to highlight areas of interests while preserving the context (e.g., a particular investigator’s direct collaborators).

It is important for a user to have the ability to narrow down the scope and reduce the complexity of the data by filtering based on her domain knowledge or interests. Such functionality facilitates users in discovering patterns and data points of interest; and it helps to focus the visual analysis process. However, it is not always easy to translate an analysis task into proper interfaces since the user may not have a well-defined hypothesis and simply wants to explore and learn the data. CollaborationViz implements a number of viewing control mechanisms–highlighting, filtering, and visual overlays–to offer services for visual navigation and visual analytics. For example, as shown in Figure 1, the centrality leaders and CTSA supported investigators are highlighted in different color and size. Highlighting helps to attract users’ attention to a small portion of highly relevant information and nodes that is directly beneficial for their analyses [29]. Further, transparency is an efficient transient highlighting techniques to dissolve the context around the object of interests. In CollaborationViz, we adjust objects’ alpha levels to render the focused objects more obvious in the display (Figure 4). Moreover, filtering and visual overlays are two other important information visualization techniques. Through filtering, we can greatly reduce the data complexity by narrowing down the scope of interests. As demonstrated in Figure 3, through filtering out non-incident nodes and edges, we can zoom in to examine the collaborative relationships of a particular node (Figure 3b), by which it eliminates the noises to the analysis problem in hand and reveals hidden patterns (e.g., the bridging and clustering effects) that were not self-evident (as in Figure 3a).

### Collaboration recommendation through link prediction

**Figure 5:**
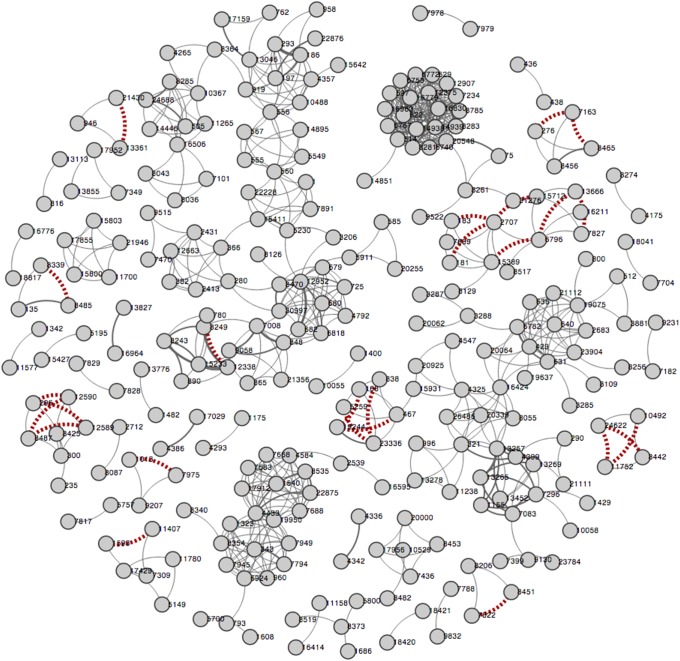
A visualization of collaboration recommendations.

Social networks such as research collaboration networks are highly dynamic, whereas new interactions among social entities are commonly manifested through additions and deletions of edges in the network. One of the main questions in studying research collaborations is how we can find promising new collaborations (new edges in the network). Such question can be tackled through applying link prediction techniques with network data. In this study, using the random walks with restarts (RWRs) method, we can accurately discover missing links (overlooked collaborations) and the links that could appear in the future (potential new collaborations). Despite the conceptual differences, the same prediction model applies to both tasks [8]. In CollaborationViz, we can depict the predicted links as dotted lines between nodes (Figure 5), which gives the user a quick overview of the predicted new collaborations and how it would affect the network dynamics.

## Discussion and Conclusion

In this study, we presented our efforts in building meaningful interactive network visualizations with theoretically based information visualization approaches to support a visual analysis process of studying research collaborations. Our result, CollaborationViz, is a novel interactive visual analytical tool for understanding social interactions among research collaborators through network analysis. The design of CollaborationViz is driven by the needs of understanding the generative mechanisms of research collaborations and helping nontechnical users in comprehending social network analysis results of RCNs in an intuitive manner. CollaborationViz provides a convenient mechanism for interactive data interrogation and exploration that enables analysts to “synthesize information and derive insight from massive, dynamic, ambiguous, and often conflicting data” and “detect the expected and discover the unexpected” [20]. The interactive visual representations in CollaborationViz make it easy for users to perceive salient aspects of the dynamics and characteristics of RCNs quickly.

CollaborationViz is designed to facilitate analytical reasoning by helping a user understand historical and current situations and enhancing user’s ability to recognize both expected and unexpected patterns in many ways. For example, as shown in Figure 2, the centripetal trend manifests in the evolution of the investigator’s collaborative relationship. The collaboration of the investigator has grown significantly, which pushes the node to move towards the center of the collaboration network at UAMS. Such a phenomenon is difficult to perceive without a visual analytical tool.

Last but not least, the ultimate goal of studying biomedical RCNs in the CTSA community is to assist administration and leaderships of research institutions to strategically allocate resources and shape policies to attain an effective, trans-disciplinary collaboration environment. CollaborationViz facilitates the dissemination of a quantitative SNA of RCNs and helps a layperson to explore, perceive, understand, and reason about complex network dynamics of the collaborative research environment. For example, a visual representation of the collaboration recommendation model [8] in CollaborationViz, as shown in Figure 5, helps to quickly identify potential new collaborations that are likely to succeed. Furthermore, the open-source nature makes CollaborationViz highly customizable and easy to be adopted by other CTSA institutions.

Efforts to develop software libraries and frameworks for network visualization have been underway in several different areas. A number of general purposed network analysis tools such as iGraph [13], NetworkX [14] and Pajek [15] have graph drawing components that provide some basic visualization functionalities. However, most of these including tools that are specialized in network (or graph) visualization (e.g., Hive plots [32] and GraphViz [30]) merely focus on graph layout algorithms to provide a static aesthetically pleasing view of the network and few of them can provide interactive user experiences (e.g., iGraph and Pajek). A few of the network visualization tools (e.g., Pajek and Gephi [31]) provide infrastructures to support time-varying and dynamic networks. And, tools such as LaNet-vi [35], Tulip [36], and Cytoscape [37] focus on visualizing large-scale networks. Further, numerous efforts have also been spurred on building domain specific network visualization to assist visual thinking and solve domain analytical problems. For example, Cytoscape [37], BioLayout [34] and Arean3D [33] are all well-known in the biomedical domain for visualizing biological networks. And, vizster [38] has been successful in allowing end-user exploration of large-scale online social networks. CollaborationViz falls into the category of a domain specific network visualization study. Nevertheless, CollaborationViz is unique in the sense that it integrates contextual information to facilitate a complete visual analysis process (e.g., analysis tasks such as observing temporal evolution of a network, studying relative importance of investigators, and predicting potential successful new collaborations) pertaining to studying and understanding of research collaboration environment. Furthermore, CollaborationViz is important for large-scale national efforts (e.g., CTSA) that promote interdisciplinary collaborations as it gives program evaluators and team science researchers a meaningful instrument to assess the impact of such programs on building a collaborative research environment and incubating new multidisciplinary collaborations.

Complex networks are commonly seen in biomedical research. Although CollaborationViz is built to specialize in exploring research collaboration networks, the underlying network visualization methods and principles can potentially be translated to other network studies such as brain connectivity networks and gene networks. Future work involves further iterations of new interactive visualization features to support more visual analysis tasks. For example, we are exploring the possibility of labeling the clusters (i.e., community structures [6]) in the network with research topics through mining grant abstracts with topic modeling methods (e.g., Latent dirichlet allocation [21]). To allow identifying relations between these topics and identify higher-level domains and disciplines, we can use a machine-understandable hierarchy that allows automated inference. Additionally, we will use more sophisticated topic relations to identify RCNs where, for example, the researchers focus on the use of a specific drug or even drugs with a specific mechanism of action (e.g., beta blockers or other antihypertensive medications). To accomplish these goals, we will use a realism-based knowledge representation system coded in Web Ontology Language (OWL) to define the topics and their relations to each other. These additions will benefit the analysis of RCNs in multiple ways, among others: a) automatically identifying existing research priorities in a network, b) identifying larger research domains that are relevant in the network, and c) automatically identifying unused research potential for research collaborations within an network.

## References

[1] CTSA Consortium: Evaluation Key Function Committee (2011) Evaluation - social network analysis. Available: https://www.ctsacentral.org/committee/evaluation-social-network-analysis. Accessed 2014 April 18.

[2] Newman ME (2001) The structure of scientific collaboration networks. Proc Natl Acad Sci USA 98: 404–409. 11149952 10.1073/pnas.021544898 PMC14598

[3] Newman ME (2004) Coauthorship networks and patterns of scientific collaboration. Proc Natl Acad Sci USA 101 Suppl 1: 5200–5205. 14745042 10.1073/pnas.0307545100 PMC387296

[4] Uddin S, Hossain L, Rasmussen K (2013) Network effects on scientific collaborations. PLoS ONE 8: e57546. 23469021 10.1371/journal.pone.0057546 PMC3585377

[5] Nagarajan R, Lowery CL, Hogan WR (2011) Temporal evolution of biomedical research grant collaborations across multiple scales–a CTSA baseline study. AMIA Annu Symp Proc 2011: 987–993. 22195158 PMC3243257

[6] Nagarajan R, Kalinka AT, Hogan WR (2013) Evidence of community structure in Biomedical Research Grant Collaborations. J Biomed Inform 46: 40–46. 22981843 10.1016/j.jbi.2012.08.002 PMC4121986

[7] Bian J, Xie M, Topaloglu U, Hudson T, Hogan W (2013) Understanding biomedicai research collaborations through social network analysis: A case study. In: Bioinformatics and Biomedicine (BIBM), 2013 IEEE International Conference on. 9–16. doi:10.1109/BIBM.2013.6732728.

[8] Bian J, Xie M, Topaloglu U, Hudson T, Eswaran H, et al.. (2014) Social network analysis of biomedical research collaboration networks in a CTSA institution. J Biomed Inform. doi:10.1016/j.jbi.2014.01.015. 10.1016/j.jbi.2014.01.015 PMC4136998 24560679

[9] Freeman LC (2000) Visualizing social networks. Journal of Social Structure 1.

[10] Heer J, Boyd D (2005) Vizster: visualizing online social networks. In: Information Visualization, 2005. INFOVIS 2005. IEEE Symposium on. 32–39. doi:10.1109/INFVIS.2005.1532126.

[11] Shen Z, Ogawa M, Teoh ST, Ma KL (2006) Biblioviz: A system for visualizing bibliography infor- mation. In: Proceedings of the 2006 Asia-Pacific Symposium on Information Visualisation - Volume 60. Darlinghurst, Australia, Australia: Australian Computer Society, Inc., APVis ’06, 93–102.

[12] Alsukhni M, Zhu Y (2012) Interactive visualization of the social network of research collaborations. In: Information Reuse and Integration (IRI), 2012 IEEE 13th International Conference on. 247–254. doi:10.1109/IRI.2012.6303017.

[13] Csardi G, Nepusz T (2006) The igraph software package for complex network research. Inter Journal Complex Systems: 1695.

[14] Hagberg AA, Schult DA, Swart PJ (2008) Exploring network structure, dynamics, and function using NetworkX. In: Proceedings of the 7th Python in Science Conference (SciPy2008). Pasadena, CA USA, 11–15.

[15] Batagelj V, Mrvar A (2003) Pajek - analysis and visualization of large networks. In: Graph Drawing Software. Springer, 77–103.

[16] W3C SVG Working Group (2011) Scalable Vector Graphics (SVG) 1.1 (Second Edition). Available: http://www.w3.org/TR/2011/REC-SVG11-20110816/. Accessed 2014 April 18.

[17] Bostock M, Ogievetsky V, Heer J (2011) D3: Data-driven documents. IEEE Trans Visualization & Comp Graphics (Proc InfoVis). 10.1109/TVCG.2011.185 22034350

[18] Twitter Inc. (2012) Bootstrap. Available: http://getbootstrap.com/. Accessed 2014 April 18.

[19] EMCA International (2013) The JSON Data Interchange Format, 1^st^ Edition. Available: http://www.ecma-international.org/publications/files/ECMA-ST/ECMA-404.pdf. Accessed 18 April 2014].

[20] Thomas JJ, Cook KA (2006) A visual analytics agenda. Computer Graphics and Applications, IEEE, 26(1), 10–13. 10.1109/mcg.2006.5 16463473

[21] Blei DM, Ng AY, Jordan MI (2003) Latent dirichlet allocation. Journal of machine Learning research, 3, 993–1022.

[22] Newman ME (2001) *Scientific collaboration networks. II. Shortest paths, weighted networks*, and centrality. Physical review E. 64(1): p. 016132. 10.1103/PhysRevE.64.016132 11461356

[23] Verlet L (1967) Computer “experiments” on classical fluids. I. Thermodynamical properties of Lennard-Jones molecules. Physical review,159(1), 98.

[24] Kobourov SG (2012) Spring embedders and force directed graph drawing algorithms. arXiv preprint arXiv: 1201.3011.

[25] Dwyer T (2009) Scalable, versatile and simple constrained graph layout. In Computer Graphics Forum (Vol. 28, No. 3, 991–998). Blackwell Publishing Ltd.

[26] Newman ME (2010) Networks: an introduction. Oxford University Press.

[27] Opsahl T, Agneessens F, Skvoretz J (2010) Node centrality in weighted networks: Generalizing degree and shortest paths. Social Networks, 32(3), 245–251.

[28] Dwork C, Kumar R, Naor M, Sivakumar D (2001) Rank aggregation methods for the web. In Proceedings of the 10th international conference on World Wide Web (613–622). ACM.

[29] Liang J, Huang ML (2010) Highlighting in information visualization: A survey. In Information Visualisation (IV), 2010 14th International Conference (79–85). IEEE.

[30] Gansner ER, North SC (2000) An open graph visualization system and its applications to software engineering. Software Practice and Experience, 30(11), 1203–1233.

[31] Bastian M, Heymann S, Jacomy M (2009) Gephi: an open source software for exploring and manipulating networks. ICWSM, 8, 361–362.

[32] Krzywinski M, Birol I, Jones SJ, Marra MA (2012) Hive plots–rational approach to visualizing networks. Briefings in bioinformatics, 13(5), 627–644. 10.1093/bib/bbr069 22155641

[33] Pavlopoulos GA, O'Donoghue SI, Satagopam VP, Soldatos TG, Pafilis E, et al.. (2008) Arena3D: visualization of biological networks in 3D. BMC Systems Biology, 2, 104. 10.1186/1752-0509-2-104 PMC2637860 19040715

[34] Enright AJ, Ouzounis CA (2001) BioLayout–an automatic graph layout algorithm for similarity visualization. Bioinformatics, 17(9), 853–854. 10.1093/bioinformatics/17.9.853 11590107

[35] Alvarez-Hamelin JI, Dall'Asta L, Barrat A, Vespignani A (2005) k-core decomposition: A tool for the visualization of large scale networks. arXiv preprint cs/0504107.

[36] Auber D, Archambault D, Bourqui R, Lambert A, Mathiaut M, et al.. (2012) The tulip 3 framework: A scalable software library for information visualization applications based on relational data.

[37] Shannon P, Markiel A, Ozier O, Baliga NS, Wang JT, et al.. (2003) Cytoscape: a software environment for integrated models of biomolecular interaction networks. Genome research, 13(11), 2498–2504. 10.1101/gr.1239303 PMC403769 14597658

[38] Heer J, Boyd D (2005) Vizster: Visualizing online social networks. In Information Visualization, 2005. INFOVIS 2005. IEEE Symposium on (32–39). IEEE.

